# TRPV4 Inhibition Improved Myelination and Reduced Glia Reactivity and Inflammation in a Cuprizone-Induced Mouse Model of Demyelination

**DOI:** 10.3389/fncel.2018.00392

**Published:** 2018-11-05

**Authors:** Meiying Liu, Xuan Liu, Lei Wang, Yu Wang, Fuxing Dong, Jian Wu, Xuebin Qu, Yanan Liu, Zhian Liu, Hongbin Fan, Ruiqin Yao

**Affiliations:** ^1^Department of Cell Biology and Neurobiology, Xuzhou Key Laboratory of Neurobiology, Jiangsu Key Laboratory of New Drug Research and Clinical Pharmacy, Xuzhou Medical University, Xuzhou, China; ^2^Department of Human Anatomy, Xuzhou Medical University, Xuzhou, China; ^3^Department of Rheumatology, Affiliated Hospital of Inner Mongolia Medical University, Hohhot, China; ^4^Department of Neurology, Affiliated Hospital of Xuzhou Medical University, Xuzhou, China

**Keywords:** demyelination, inflammation, TRPV4, NF-κB, oligodendrocyte

## Abstract

The inhibition of demyelination and the promotion of remyelination are both considerable challenges in the therapeutic process for many central nervous system (CNS) diseases. Increasing evidence has demonstrated that neuroglial activation and neuroinflammation are responsible for myelin sheath damage during demyelinating disorders. It has been revealed that the nonselective cation channel transient receptor potential vanilloid 4 (TRPV4) profoundly affects a variety of physiological processes, including inflammation. However, its roles and mechanisms in demyelination have remained unclear. Here, for the first time, we found that there was a significant increase in TRPV4 in the corpus callosum in a demyelinated mouse model induced by cuprizone (CPZ). RN-1734, a TRPV4-antagonist, clearly alleviated demyelination and inhibited glial activation and the production of tumor necrosis factor α (TNF-α) and interleukin 1β (IL-1β) without altering the number of olig2-positive cells. *In vitro*, RN-1734 treatment clearly inhibited the influx of calcium and decreased the levels of IL-1β and TNF-α in lipopolysaccharide (LPS)-activated microglial cells by suppressing NF-κB P65 phosphorylation. Apoptosis of oligodendrocyte induced by LPS-activated microglia was also alleviated by RN-1734. The results suggest that activation of TRPV4 in microglia is involved in oligodendrocyte apoptosis through the activation of the NF-κB signaling pathway, thus revealing a new mechanism of CNS demyelination.

## Introduction

Demyelination is the process of the loss of myelin and is one of the important pathological features of many central nervous system (CNS) diseases, such as multiple sclerosis (MS), ischemic stroke and Alzheimer’s disease (AD; Holmøy and Hestvik, 2008; Carmeli et al., 2013; Wang et al., 2016). Demyelination is always concomitant with glial activation and lymphocyte/macrophage infiltration. Activated glial cells excessively release massive amounts of cytokines/chemokines, which cause damage to oligodendrocyte and ultimately lead to severe demyelination (Emery et al., 2006; Suzuki et al., 2010; Petković et al., 2017). The inflammatory and activated milieu surrounding the demyelinated lesions compromises and limits the process of remyelination (Franklin and Goldman, 2015). Therefore, regulation of the inflammatory microenvironment during the progression of demyelination would be beneficial to remyelination. However, the complex mechanisms involved in oligodendrocyte damage and glial activation have not been fully elucidated.

Transient receptor potential vanilloid 4 (TRPV4), a nonselective cation channel, belongs to the group of TRP cation channels. Previous studies have reported that TRPV4 is expressed in neurons, endothelial cells, microglia and astrocytes (Benfenati et al., 2007; Shibasaki et al., 2007; Harraz et al., 2018). Recently, it was found that TRPV4 is also expressed in oligodendrocyte precursor cells (OPCs) and promotes OPC proliferation (Ohashi et al., 2018). TRPV4 is activated by a broad range of stimuli, such as physical factors, endogenous and exogenous chemical factors and low pH, and affects a variety of physiological processes, including inflammation (Garcia-Elias et al., 2014; Ishikura et al., 2015). Significant infrasound-induced astrocytic and microglial activation promotes an increase inTRPV4 expression and the release of the proinflammatory cytokines interleukin-1β (IL-1β) and tumor necrosis factor α (TNF-α), which are responsible for infrasound-induced neuronal apoptosis (Shi et al., 2013). Changes in osmolarity activate TRPV4, which leads to an increase in proinflammatory cytokines IL-1β and IL-6 in intervertebral disc cells (Walter et al., 2016). Since CNS demyelination is accompanied by glial activation and inflammation, we suggest that the activation of TRPV4 and the release of proinflammatory cytokines in microglia may be involved in oligodendrocyte apoptosis and demyelination.

The cuprizone (bis-cyclohexanone oxaldihydrazone, CPZ)-induced demyelination model is well established and is used to investigate demyelination and remyelination in rodents (Stidworthy et al., 2003; Skripuletz et al., 2011). CPZ feeding for 5 weeks induced glial cell activation and oligodendrocyte death, which resulted in demyelination, in C57BL/6 mice (Hashimoto et al., 2017). In this study, we found that TRPV4 was increasingly expressed in a CPZ-induced demyelination mouse model and that the use of its antagonist partially prevented demyelination, glia reactivity and inflammation. The results were further confirmed *in vitro*, and LPS-induced TRPV4 activation in microglial cells increased the levels of IL-1β and TNF-α by activating the NF-κB signaling pathway and caused oligodendrocyte apoptosis. This study suggests that inhibition of TRPV4 may protect the myelin sheath from degeneration by alleviating inflammation in the CPZ-induced demyelination model.

## Materials and Methods

### Animals and Drug Treatment

C57BL/6 male mice (8 weeks old) were purchased from Laboratory Animal Center, Xuzhou Medical University, Jiangsu, China, and housed with free access to food and water (12-h light/dark cycle) under strict hygienic conditions. All procedures in this study were in accordance with the guidelines of the National Institutes of Health (NIH) Guide for the Care and Use of Laboratory Animals, complied with Chinese legislation and were approved by Institutional Animal Care and Use Committee of Xuzhou Medical University (No. 201602). We made all efforts to minimize animal suffering and reduce the number of animals used.

For drug treatment, C57BL/6 mice were divided into four groups: the normal control group (Ctrl), the CPZ group, the CPZ- and vehicle-treated group (vehicle) and the CPZ- and RN-1734-treated group (RN-1734). The control group mice were fed standard diets. The mice in the CPZ group were fed with 0.2% CPZ (Sigma-Aldrich, USA) for 5 weeks to induce demyelination (Skripuletz et al., 2011). The mice in the vehicle group and the RN-1734 group were injected with DMSO or RN-1734 during CPZ feeding. Briefly, mice were placed in a stereotaxic apparatus (RWD Life Science Co., China) after isoflurane anesthesia, and 26-gauge stainless steel guide cannulas (RWD Life Science Co., China) were chronically implanted into the lateral ventricle at the following coordinates: anterior-posterior +1.0, medial-lateral 0.0 and dorsal-ventral −3.0 (Paxinos and Franklin, 2001). A 28-gauge dummy cannula was inserted into the guide cannula if the guide cannula was clogged. Daily infusion of 0.5 μl of vehicle (5%DMSO in 0.9% NaCl) or RN-1734 (10 μM in 5% DMSO and 0.9% NaCl, Sigma-Aldrich) was performed with a microinjector pump (RWD Life Science Co., China) at a rate of 0.1 μl/min for 5 weeks. All the experimental procedures were based on previous reports in the literature (Fichna et al., 2012).

### Histology and Immunofluorescence

Mice (*n* = 4 for each group) were anesthetized with 10% chloral hydrate, transcardially perfused with 0.9% normal saline, and then fixed with cold 4% paraformaldehyde (PFA). Brains were carefully removed, postfixed in 4% PFA for 12 h, and dehydrated in 30% sucrose solution for 48 h at 4°C. Coronal sections of 20 μm were prepared by cryosectioning (Leica Microsystems, Germany) and stored at −80°C. For immunofluorescence, the brain sections were blocked with 10% goat serum and 0.3% Triton X-100 in 0.01 mol/L phosphate buffered saline (PBS) for 40 min at 37°C, followed by incubation with primary antibodies overnight at 4°C. The primary antibodies used were mouse anti-2′,3′-cyclic nucleotide 3′ phosphodiesterase antibody (CNP; IgG, 1:1,000, Santa Cruz, Santa Cruz, CA, USA), rabbit anti-TRPV4 and rabbit anti-olig2 antibodies (both IgG, 1:200, Abcam Cambridge, UK), rabbit anti-glial fibrillary acidic protein antibody (GFAP; IgG, 1:500, Abcam, USA), rabbit anti-ionized calcium binding adaptor molecule-1 antibody (Iba-1; IgG, 1:1,000, Cell Signaling Technology/CST, Danvers, MA, USA) and rabbit anti-cleaved caspase3 antibody (IgG, 1:300, CST, Danvers, MA, USA). The sections were rinsed three times with PBS and treated with IFKine Red donkey anti-mouse IgG, IFKine Green donkey anti-mouse IgG, IFKine Red donkey anti-rabbit IgG and IFKine Green donkey anti-rabbit IgG (1:500; Abbkine, USA) overnight at 4°C. 4′,6-Diamidino-2-phenylindole (DAPI, Beyotime Biotechnology) was used for nuclear staining. Finally, the sections were washed with PBS and mounted with anti-fade mounting medium (Beyotime Biotechnology). Images were captured using a fluorescence microscope (Olympus BX43, Japan). The integrated optical density (IOD) at the same levels from three sections per animal was measured using Image-ProPlus 6.0 software.

### Western Blot Analysis

Mice (*n* = 3 for each group) were anesthetized with 10% chloral hydrate and quickly sacrificed, and the corpus callosum was immediately dissected and stored at −80°C. The samples of corpus callosum were lysed in radio immunoprecipitation assay (RIPA) lysis buffer and the protease inhibitor phenylmethylsulfonyl fluoride (PMSF; Beyotime Biotechnology). Then, they were homogenized and centrifuged at 12,000× *g* for 15 min at 4°C to collect the supernatant for protein detection. Equal amounts of protein (80 μg) were separated with sodium dodecyl sulfate-polyacrylamide gel electrophoresis (SDS-PAGE) and transferred onto nitrocellulose (NC) membranes (Bio-Rad, CA, Hercules, USA). The membranes were blocked with 5% bovine serum albumin (BSA) for 2 h at room temperature and incubated with mouse anti-2′,3′-cyclic nucleotide 3′phosphodiesterase antibody (CNP; IgG, 1:1,000, Santa Cruz, CA, USA), rabbit anti-p-NF-κB P65 (1:500, CST, USA), rabbit NF-κB P65 (1:500, CST, USA) and rabbit anti β-actin (IgG, 1:1,000, Santa Cruz, USA) primary antibodies overnight at 4°C. After washing three times in washing buffer, the membranes were incubated with the corresponding conjugated goat anti-rabbit or goat anti-mouse IgG-horseradish peroxidase (HRP) secondary antibodies (1:1,000, Santa Cruz, USA) for 2 h at room temperature. Then, signals were visualized with an Odyssey infrared imaging system (LI-COR, Lincoln, NE, USA), and the densitometric values of the bands were quantified with ImageJ software. All experiments were repeated at least three times.

### Electron Microscopy

Preparation for transmission electron microscopy (TEM) was performed as previously described (Qu et al., 2015). Briefly, mice (*n* = 3 for each group) were anesthetized and transcardially perfused with2% glutaraldehyde (Gla) and 2.5% PFA in 0.1 mol/L PBS. Samples of corpus callosum were immediately extracted and postfixed in 3% Gla and 1.5% PFA at 4°C overnight and transferred to 1% osmium tetroxide for 1 h at room temperature. After dehydrating with ascending ethanol concentrations, the 1-mm^3^ blocks were embedded in Epon618, and then 1 μm semi thin sections were cut for toluidine blue staining. Ultrathin sections (70 nm) were cut from the resin-embedded samples and stained with uranyl acetate and lead citrate prior to examination by TEM (FEI Tecnai™ G2 T12, USA). The images were analyzed with TEM Imaging & Analysis (TIA) software. Ten visual fields were chosen randomly, and at least 100 axons were measured. The axonal diameter (d) was defined as the shortest distance across the center of an axon. The axonal diameter plus the total myelin sheath thickness on both sides was defined as the fiber diameter (D). The G-ratio was calculated using the d/D ratio.

### Primary Microglia and Oligodendrocyte Culture and Drug Treatment

Primary microglia and oligodendrocyte culture was performed as previously described (Lai et al., 2017). Cells derived from the cortex of neonatal Sprague-Dawley (SD) rats (postnatal day 1–3) purchased from Laboratory Animal Center, Xuzhou Medical University, were placed into DMEM/F12 (HyClone) containing 10% fetal bovine serum (FBS, Clark). Microglia were purified from 8-day-old mixed glial cultures by shaking the floating fraction on a rotary shaker for 1 h at 37°C, and then OPCs were harvested by collecting the cell suspension of the mixed glial cell cultures after shaking the flasks on a horizontal orbital shaker for 18 h at 37°C. Then, microglial cells were pelleted at 600× *g* for 5 min and resuspended in high-glucose DMEM (HyClone) with 10% FBS at 37°C in a humid atmosphere with 5% CO_2_. The medium was changed every 2 days. To observe whether TRPV4 activation causes the release of proinflammatory factors and oligodendrocyte damage, the microglial cells were divided into a normal group and a lipopolysaccharide (LPS) group. LPS-treated microglial cells were further divided into a LPS group, a vehicle (DMSO treatment) group and a RN-1734 group. The cells were treated with 1 μg/ml LPS (Sigma) or RN-1734 (10 μM) for 3 h (Shin et al., 2010; Kato and Morita, 2011). Next, the cells were triple-washed with DMEM medium to remove LPS, and then incubated in DMEM with or without RN1734 for 24 h. Finally, the cells were collected for TRPV4 immunocytostaining, and the supernatant was collected separately for enzyme-linked immunosorbent assay (ELISA) or for the oligodendrocyte apoptosis experiment. The supernatant of the microglial cells was mixed with DMEM/F12 at a ratio of 1:1. The mixed medium without LPS was used for the control (Ctrl) group, the medium with LPS only was used for the conditional medium (CM) group, and the media with LPS and DMSO or RN-1734 were used for the CM-vehicle group and the CM-RN1734 group, respectively. After oligodendrocytes grew for 2 days in 6-well plates in DMEM/F12 containing 10% FBS, the differentiation medium was replaced with the described Ctrl or different CM media for 24 h. Then, the cells were harvested for apoptosis analysis and western blot analysis separately.

The OPCs were pelleted at 600× *g* for 5 min and then resuspended in DMEM/F12 (HyClone) containing 10 ng/ml recombinant human fibroblast (bFGF), 10 ng/ml recombinant human platelet-derived growth factor-AA (PDGF-AA; Peprotech, Rocky Hill, NJ, USA), 1% N2 and 2% B27 (Invitrogen Corp, Carlsbad, CA, USA). The cells were inoculated on poly-L-lysine-coated 6-well cell culture plates at 1 × 10^5^ cells/ml, and the medium was changed every 2 days. For oligodendrocyte production, after the OPCs grew for 4 days, PDGF-AA and bFGF were excluded from the OPC medium, and 10% FBS was added to differentiate the OPCs into oligodendrocytes.

### Terminal Deoxynucleotidyl Transferase dUTP Nick End Labeling (TUNEL) Staining

A terminal deoxynucleotidyl transferase dUTP nick end labeling (TUNEL) fluorescein kit (Roche, Switzerland) was used to assess apoptotic cells according to the manufacturer’s protocol. Briefly, OPCs were fixed with 4% PFA for 20 min, treated with 1% Triton X-100 for 5 min at room temperature and then incubated with TUNEL reaction fluid for 1 h at 37°C while protected from light. After incubation with DAPI for 10 min, the cells were washed with PBS. Five microscopic fields were randomly selected, and the TUNEL-positive cells were counted. The apoptotic rate was calculated using the following formula: apoptotic rate = TUNEL positive cells/total cells per field (DAPI positive nucleus) × 100%.

### ELISA

The IL-1β and TNF-α levels in mouse serum and cell culture supernatant were tested using mouse IL-1β or TNF-α instant ELISA test kits (eBiosciences). All procedures were carried out according to the manufacturer’s protocol. The absorbance at 450 nm was measured with a spectrophotometer (San Francisco, CA, USA), and the absorbance data of the IL-1β and TNF-α samples were determined.

### Measurement of Intracellular Ca^2+^

Microglial cells were washed twice with Hank’s balanced salt solution (HBSS) and incubated in a final concentration of 2 μM Rhod-2 AM (Abcam) for 30 min at room temperature. After two washes with pre-warmed HBSS, the cells were further incubated for 30 min at 37°C, and images were taken with a Zeiss Axioskop 40 microscope (Carl Zeiss, Oberkochen, Germany) with the following settings: excitation laser = 405 nm and emission gate center = 519 nm. The mean fluorescence intensity (MFI) of Ca^2+^ was measured with Image-ProPlus 6.0 software. Five fields/well and approximately 15–20 cells per field were examined in each group (*n* = 5).

### Statistical Analysis

All of the statistical data were analyzed with SPSS software version 19.0 and are shown as the means ± SEM. *T*-tests or one-way analysis of variance (ANOVA) followed by Newman-Keuls or Tukey’s HSD post tests were used for comparisons between two groups or multiple groups, respectively. Statistical significance was set at *P* < 0.05.

## Results

### TRPV4 Was Activated in the CPZ-Induced Demyelination Mouse Model

**Figure 1:**
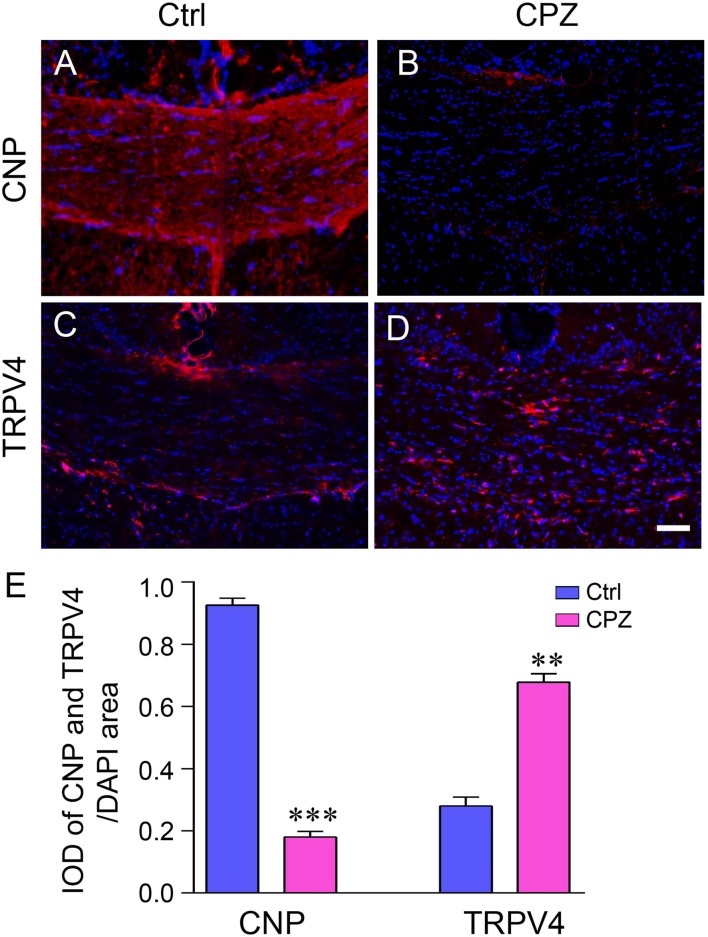
The expression of transient receptor potential vanilloid 4 (TRPV4) was increased in the cuprizone (CPZ)-treated mice. Immunofluorescence staining of 2′,3′-cyclic nucleotide 3′ phosphodiesterase antibody (CNP) **(A,B)** and TRPV4 **(C,D)** in the corpus callosum of the control (Ctrl) group and the CPZ group. Scale bar = 20 μm. **(E)** Integrated optical density (IOD) analysis of CNP- and TRPV4-positive cells. *n* = 4 per group. The data are shown as the mean ± SEM. **P < 0.01, ***P < 0.001 vs. the Ctrl group.

The mature oligodendrocyte marker CNP was used to examine the extent of demyelination in immunofluorescence staining. The mice treated with CPZ for 5 weeks showed severe myelin sheath loss (Figures 1A,B). The IOD of CNP-positive cells in the corpus callosum in the CPZ group was significantly decreased compared to that in the Ctrl group (Figure 1E; *P* < 0.001). This indicates that the demyelination mouse model was successfully established in this study. In contrast, the immunofluorescence signal of TRPV4 was stronger in the CPZ-treated mice (Figures 1C,D) and the IOD of TRPV4 in the corpus callosum was clearly increased in the CPZ group compared to the Ctrl group (Figure 1E; *P* < 0.01).

### TRPV4 Was Involved in Demyelination in the CPZ-Induced Demyelination Mouse Model

**Figure 2:**
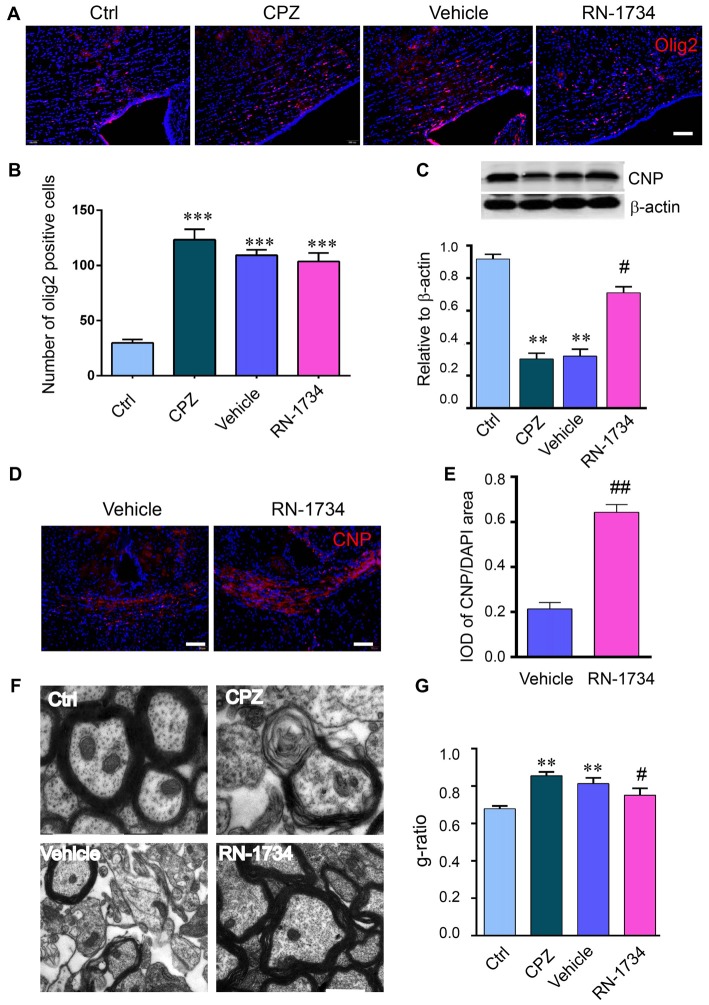
Inhibition of TRPV4 activity alleviated demyelination induced by CPZ. **(A,B)** Images and quantitative analysis of olig2 immunostaining cells in the Ctrl, CPZ, vehicle-treated (vehicle) and RN-1734-treated (RN-1734) groups (*n* = 4). Scale bar = 50 μm. **(A–C)** Western blot analysis of the corpus callosum CNP in the control, CPZ, vehicle and RN-1734 groups (*n* = 3). **(B–E)** Immunofluorescence and quantitative analysis of CNP-positive cells in the vehicle group and the RN-1734 group (*n* = 4). Scale bar = 50 μm. **(D–F)** Representative images from electron microscopy of the corpus callosum in the control, CPZ, vehicle and RN-1734 groups. **(E–G)** Quantitative analysis of the *g-*ratio of the myelin sheath (*n* = 3). Scale bar = 500 nm. The data are shown as the mean ± SEM. **P < 0.01, ***P < 0.001 vs. the Ctrl group; ^#^*P* < 0.05, ^##^*P* < 0.01 vs. the vehicle group.

To investigate whether TRPV4 is involved in demyelination during CPZ exposure, we inhibited TRPV4 activity using the selective antagonist RN-1734. CPZ caused a clear increase in the number of Olig2-positive cells (Figures 2A,B; *P* < 0.001) and a decrease in CNP protein in the corpus callosum compared to that in the Ctrl group (Figures 2A–C; *P* < 0.01). RN-1734 significantly reversed the decrease in CNP protein induced by CPZ treatment, but not affected the number of Olig2-positive cells (Figures 2A,B; *P* < 0.05). The IOD of CNP-positive cells was also increased in the RN-1734 group compared to the vehicle group (Figures 2B–E; *P* < 0.01). Ultrastructure analyses revealed a severe demyelination of axons in the mice fed CPZ, and RN-1734 treatment improved myelination (Figure 2F). Due to the thickness of the myelin sheath, the *g*-ratio of the myelin sheath in the CPZ group was increased compared to that in the Ctrl group (Figures 2F,G; *P* < 0.01), while the *g*-ratio was decreased significantly in the RN-1734 group compared to the vehicle group (Figures 2F,G; *P* < 0.05). These data suggested that TRPV4 activation was involved in the CPZ-induced demyelination.

### Inhibition of TRPV4 Activity Suppressed Glial Activation and Proinflammatory Cytokine Production

**Figure 3:**
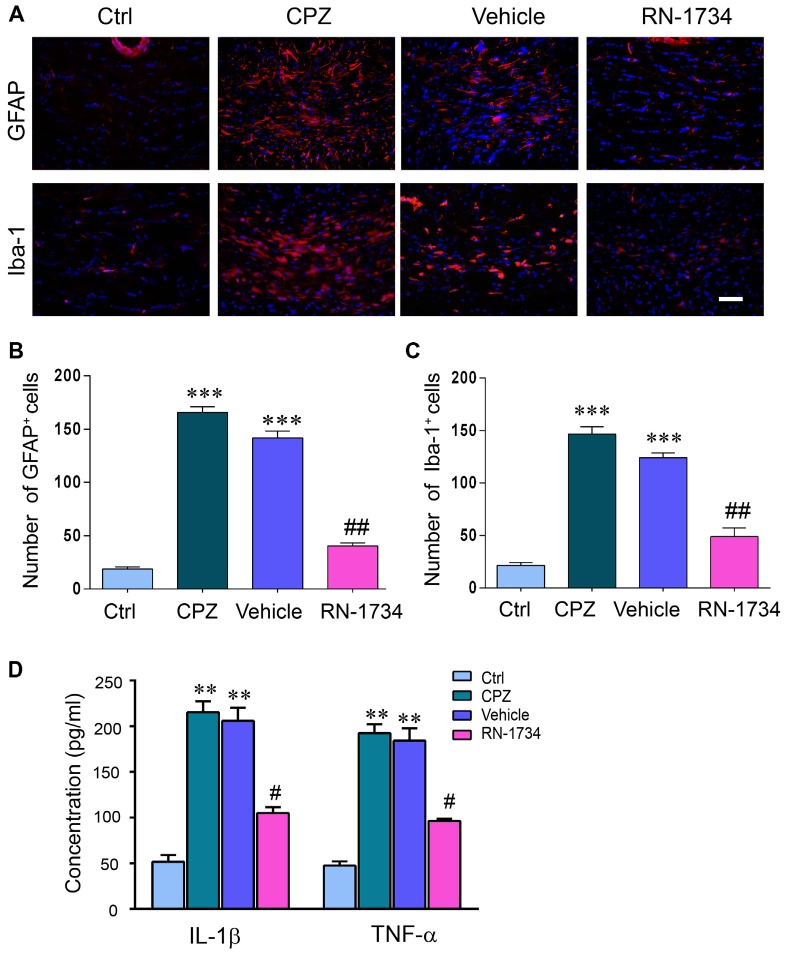
TRPV4 activation was involved in glial activation and proinflammatory cytokines release. **(A)** Representative glial fibrillary acidic protein antibody (GFAP) and Iba-1 immunofluorescent images for the corpus callosum. Scale bar = 20 μm. **(B,C)** Quantitative analysis of GFAP- and Iba-1-positive cell numbers. The increased numbers of GFAP- and Iba-1-positive cells were reversed by the TRPV4 inhibitor RN-1734 (*n* = 4). **(D)** Protein levels of tumor necrosis factor alpha (TNF-α) and interleukin 1beta (IL-1β) were detected by enzyme-linked immunosorbent assay (ELISA) (*n* = 3). The data are expressed as the mean ± SEM. **P < 0.01, ***P < 0.001 vs. the Ctrl group; ^#^*P* < 0.05, ^##^*P* < 0.01 vs. the vehicle group.

CNS demyelination is accompanied by glial activation and inflammation. We examined the activation of astrocytes and microglia in CPZ-treated mice with GFAP and Iba-1 immunofluorescence staining. The numbers of both GFAP-positive and Iba-1-positive glial cells were markedly increased in the CPZ group compared to the Ctrl group (Figures 3A–C; *P* < 0.001); however, inhibition of TRPV4 activation with RN-1734 decreased the numbers of GFAP-positive and Iba-1-positive glial cells compared to that in the vehicle group (Figures 3A–C; *P* < 0.01). In addition, the levels of the proinflammatory cytokines IL-1β and TNF-α were also significantly enhanced in the CPZ-treated mice (Figure 3D; *P* < 0.01), but they were reduced in the RN-1734 group compared with the vehicle group (Figure 3D; *P* < 0.05).

### TRPV4 Activation Increased the Release of Proinflammatory Cytokines by Activating the NF-κB Signaling Pathway

**Figure 4:**
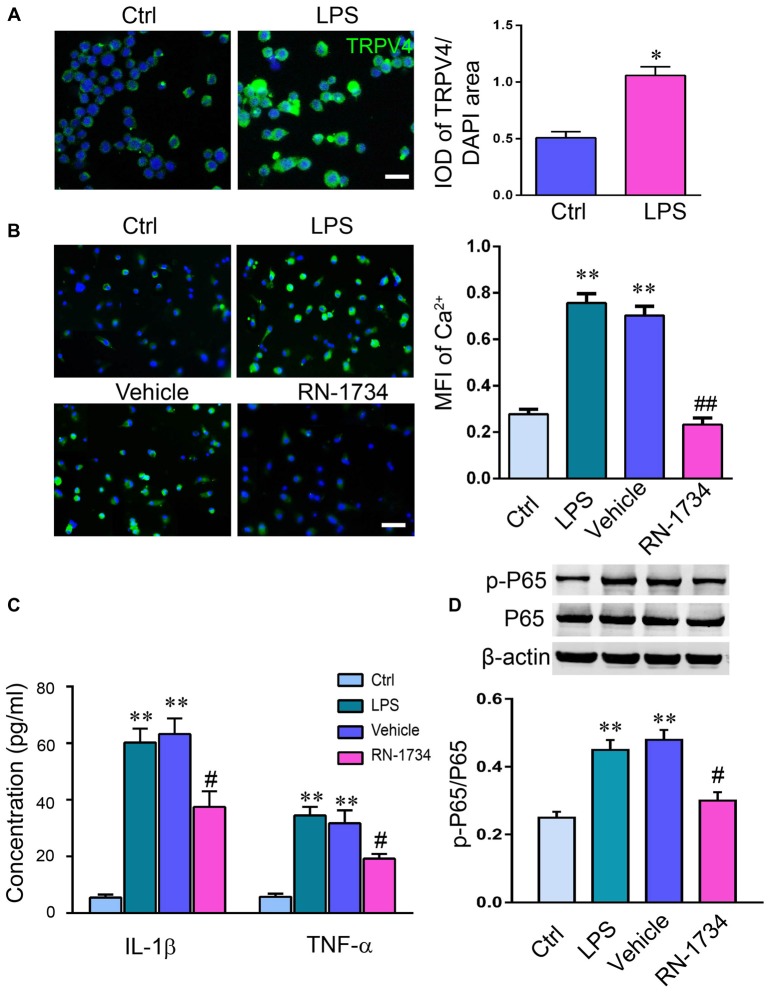
TRPV4 activation increased the release of proinflammatory cytokines by activating the NF-κB signaling pathway. **(A,B)** Lipopolysaccharide (LPS) increased the IOD of TRPV4-positive microglial cells. **(C)** The level of intracellular Ca^2+^ in microglia was measured with Rhod-2 AM. The mean fluorescence intensity (MFI) of Ca^2+^ was elevated by LPS stimulation, and RN-1734 treatment reversed the increase in the MFI of Ca^2+^. **(D)** The concentration of TNF-α and IL-1β detected by ELISA in the Ctrl, CPZ, vehicle and RN-1734 groups. *n* = 5 per group. The data are expressed as the mean ± SEM. *P < 0.05, **P < 0.01 vs. the Ctrl group; ^#^*P* < 0.05, ^##^*P* < 0.01 vs. the vehicle group.

To clarify that the release of proinflammatory cytokines is related to the activation of TRPV4 in glial cells, we measured the activation of TRPV4 and the levels of IL-1β, TNF-α and p-NF-κB P65 in cultured microglia. LPS stimulation increased the IOD of TRPV4-positive cells in microglia compared to that of the Ctrl group (Figure 4A; *P* < 0.05). The MFI of Ca^2+^ was elevated in LPS-treated microglial cells compared to Ctrl microglial cells (Figure 4B; *P* < 0.01), and RN-1734 treatment reversed the increase of MFI of Ca^2+^ induced by LPS (Figure 4B; *P* < 0.01), suggesting that the influx of calcium mediated by TRPV4 was inhibited by RN-1734. LPS stimulation caused an increase in IL-1β and TNF-α levels (Figure 4C; *P* < 0.01), and RN-1734 treatment decreased the production of IL-1β and TNF-α (Figure 4C; *P* < 0.05). In addition, we found that LPS activated the NF-κB signaling pathway; however, RN-1734 significantly decreased the expression of p-NF-κB P65 compared with that in the vehicle group (Figure 4D; *P* < 0.05). This indicates that TRPV4 activation caused the release of proinflammatory cytokines by activating the NF-κB signaling pathway.

### TRPV4 Activation in Microglia Resulted in Oligodendrocyte Apoptosis *in vitro*

**Figure 5:**
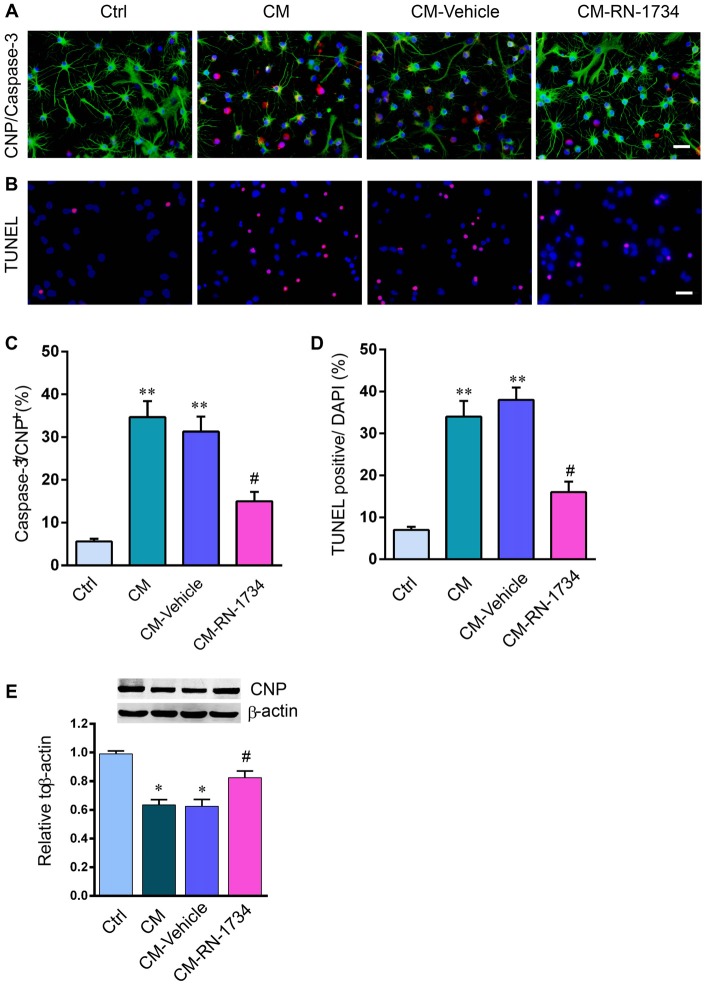
TRPV4 activation in microglia was involved in oligodendrocyte apoptosis *in vitro*. **(A,B)** Representative pictures of CNP and cleaved-caspase 3 immunofluorescence double staining and Terminal Deoxynucleotidyl TransferasedUTP Nick End Labeling (TUNEL) staining in the Ctrl, conditional medium (CM), CM-vehicle and CM-RN-1734 groups. **(C,D)** Quantitative analysis of the percentage of cleaved-caspase 3-positive cells and TUNEL-positive cells. **(E)** Western blot analysis of oligodendrocyte CNP in the ctrl, CPZ, vehicle and RN-1734 groups. *n* = 5 per group. The data are expressed as the mean ± SEM. *P < 0.05, **P < 0.01 vs. the Ctrl group; ^#^*P* < 0.05 vs. the vehicle group.

We used the conditioned medium of LPS-stimulated microglia to incubate oligodendrocytes and then detected the apoptosis of oligodendrocytes. The percentage of cleaved-caspase 3-positive oligodendrocytes was markedly increased in the CM group compared to the Ctrl group (Figures 5A,C; *P* < 0.01). RN-1734 significantly decreased the percentage of cleaved-caspase 3 positive cells compared to the percentage in the vehicle group (Figures 5A,C; *P* < 0.05). TUNEL staining also showed that CM treatment increased the apoptotic rate of oligodendrocytes compared to that of the Ctrl group (Figures 5B,D; *P* < 0.01), and RN-1734 treatment reversed the increase in the apoptotic rate of oligodendrocytes induced by CM (Figures 5B,D; *P* < 0.05). In addition, CM-induced decreases in CNP were alleviated by RN-1734 treatment (Figure 5E; *P* < 0.05). The results indicate that TRPV4 activation in microglia resulted in oligodendrocyte apoptosis.

## Discussion

Previous studies have confirmed that TRPV4 is abundantly expressed in the CNS and plays a key role in many CNS diseases, such as ischemia, seizure and epilepsy (Butenko et al., 2012; Hunt et al., 2012; Chen et al., 2016). One of the mechanisms of TRPV4 involvement in the pathological process may be related to its role in increasing the release of proinflammatory cytokines (Shi et al., 2013; Walter et al., 2016). Here, for the first time, we report that TRPV4 activation in microglia results in the release of proinflammatory cytokines through the activation of NF-κB signaling pathway, which causes oligodendrocyte apoptosis *in vitro* and demyelination in the corpus callosum of a CPZ-induced demyelinated mouse model. This study reveals a new mechanism by which TRPV4-mediated inflammation is involved in myelin sheath damage in the CNS and may provide a possible therapeutic target for demyelinating diseases.

The CPZ model has been extensively used to study de- or remyelination in the CNS (Liu et al., 2017). In line with previous reports, we successfully established a demyelination mouse model by feeding mice 0.2% CPZ for 5 weeks. Furthermore, severe demyelination was accompanied by clear microglial and astrocytic activation (Liu et al., 2017; Patergnani et al., 2017; Yu et al., 2018). In addition, we also observed a significant increase in the expression of TRPV4 in the corpus callosum of CPZ-treated mice, suggesting that TRPV4 activity may be related to myelin sheath damage in mice with CPZ-induced demyelination.

It has been reported that neuronal, glial, epithelial and secretory cells express TRPV4. A variety of factors, including osmolarity, arachidonic acid, temperature, mechanical stress and pH stimuli, can activate theTRPV4 channel (White et al., 2016; Rajasekhar et al., 2017). Recently, Ohashi et al. (2018) reported that TRPV4 is functionally expressed in OPCs and its activation significantly increased the proliferation of OPCs without affecting their migration and differentiation, however, inhibition of TRPV4 did not affect the proliferation of OPCs. Unfortunately, the study was performed *in vitro* conditions. The effects of TRPV4 on oligodendrogenesis *in vivo* remain unclear. In our present study, we found that TRPV4 inhibition did not alter the number of olig2-positive OPCs in the corpus callosum of CPZ mice, suggesting that the improvement of the remyelination may be not due to the proliferation and differentiation by TRPV4 inhibition. More specific and strict *in vivo* experiments should be performed to reveal the direct impacts of the TRPV4 on oligodendrocytes in various physiological and demyelination conditions.

TRPV4 activation induces Ca^2+^ influx and increases [Ca^2+^]_i_ in various cell types, which is associated with inflammatory and metabolic responses, neuropathic pain and many CNS disorders (Nilius and Voets, 2013; Chen et al., 2015; Tang et al., 2015). TRPV4 channels mediate Ca^2+^ influx into astrocytes and neurons and glutamate accumulation during acute stroke, and knockout of Trpv4 attenuates neuronal calcium elevation (Rakers et al., 2017). TRPV4 activation impairs the dendritic branching of hippocampal newborn neurons (Tian et al., 2017). Blocking Ca^2+^ influx through TRPV4 channels with the TRPV4 inhibitor HC067047 reduces the death of cultured dorsal root ganglion neurons and alleviates paclitaxel-induced neuropathy (Boehmerle et al., 2018). These data suggest that the TRPV4 channel may be a potential therapeutic target in clinical studies.

Several studies have indicated that TRPV4 activation in glial cells increases the production and release of inflammatory cytokines. A subpopulation of astrocytes and microglia express functional TRPV4. It has been reported that a TRPV4 agonist (4αPDD) combined with LPS diminishes microglial release of TNF and galectin-3 due to Ca^2+^-dependent suppression of microglial activity (Konno et al., 2012). In contrast, infrasound increases TRPV4 expression, which enhances the release of the proinflammatory cytokines IL-1β and TNF-α. TRPV4 knockdown or inhibition decreases the levels of IL-1β and TNF-α in cultured glial cells, attenuates neuronal apoptosis, and reduces NF-κB nuclear translocation (Shi et al., 2013). Osmolarity-activated TRPV4 also increases the production of IL-1β and IL-6 in intervertebral disc cells (Walter et al., 2016). In the current study, we confirmed that inhibition of TRPV4 activity with RN-1734 suppressed microglial and astrocytic activation and decreased the production of IL-1β and TNF-α in mice with CPZ-induced demyelination. In addition, LPS stimulation also increased the expression of TRPV4 in cultured microglia. The levels of IL-1β and TNF-α markedly decreased, and Ca^2+^ signaling elicited by LPS was attenuated back to control levels by RN-1734. Furthermore, the NF-κB signaling pathway was also inhibited by the TRPV4 inhibitor. These data suggest that the release of proinflammatory cytokines through activation of the NF-κB signaling pathway was mediated by TRPV4. Previous studies have indicated that TNF-α and IL-1β are closely related as mediators of demyelination pathology (Musumeci et al., 2011; Ramirez-Ramirez et al., 2013; Mori et al., 2014). Thus, we hypothesized that TRPV4 activation and the release of proinflammatory cytokines may be involved in oligodendrocyte damage and demyelination.

To investigate whether TRPV4 activation in microglia caused oligodendrocyte apoptosis, we used medium derived from LPS-stimulated microglia to treat oligodendrocytes. The results showed that inhibition of TRPV4 activation significantly decreased the apoptosis of oligodendrocytes induced by the CM of activated microglia and increased the expression of CNP, suggesting that TRPV4 activation in microglia resulted in oligodendrocyte apoptosis. Since both microglia and astrocytes have a crucial role in demyelination and TRPV4 was activated in the two type *in vivo*, unfortunately, we only looked to its effect on microglia, and not a direct effect in astrocytes, which should be addressed in the next studies.

**Figure 6:**
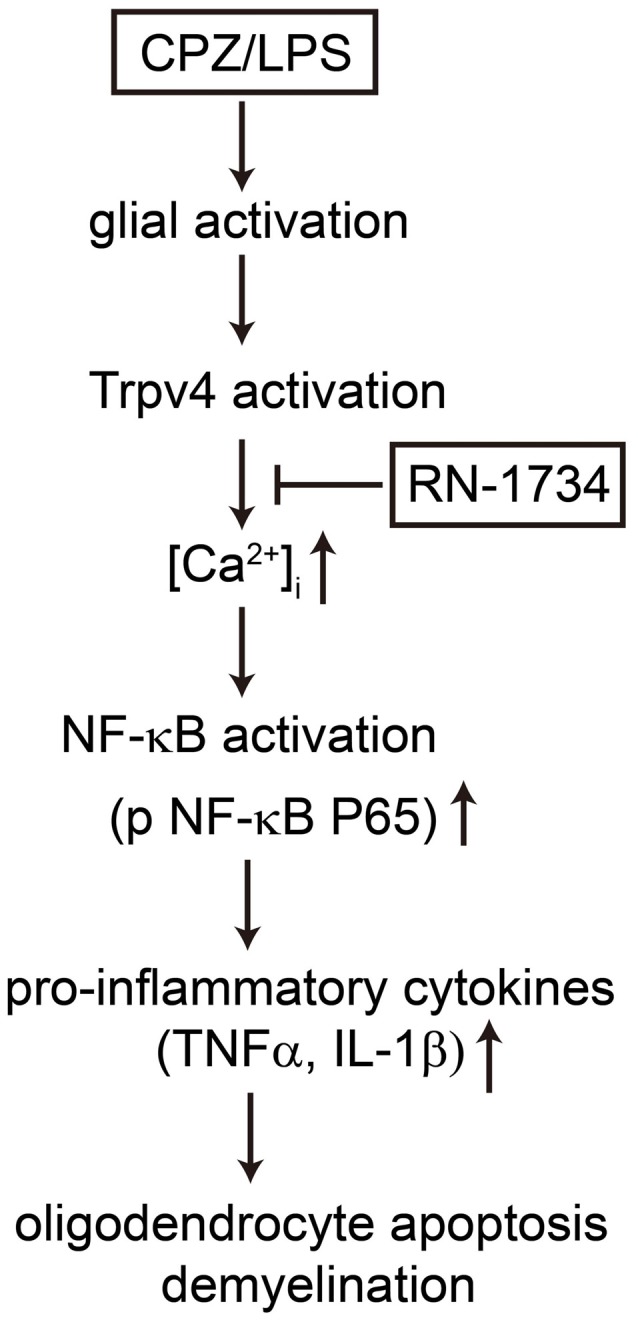
Possible mechanisms by which TRPV4 activation caused oligodendrocyte apoptosis and demyelination. TRPV4 activation induced by CPZ or LPS increases Ca^2+^ influx and glial activation, which promotes the release of TNF-α and IL-1β by activating the NF-κB signaling pathway. The production of proinflammatory cytokines results in oligodendrocyte apoptosis and demyelination. The TRPV4 inhibitor RN-1734 inhibits the NF-κB signaling pathway, decreases the level of proinflammatory cytokines and rescues oligodendrocyte damage and demyelination.

In summary, our present study provides evidence that TRPV4 activation increases the release of proinflammatory cytokines by activating the NF-κB signaling pathway. Inhibition of TRPV4 activation alleviated oligodendrocyte apoptosis by suppressing the inflammatory response of glial cells (Figure 6). Therefore, TRPV4 channels may represent a promising novel target to ameliorate neuroinflammation-mediated demyelination diseases.

## Author Contributions

ML and XL performed the experiments, collected and analyzed the data, and prepared the manuscript. LW performed the experiments, provived the data and revised the article. YW, FD, JW and XQ performed the experiments and collected the data. YL and ZL collected and analyzed data. HF and RY designed the study, provided financial support and significantly edited the manuscript. All the authors approved the final approval of the manuscript.

## Conflict of Interest Statement

The authors declare that the research was conducted in the absence of any commercial or financial relationships that could be construed as a potential conflict of interest.
